# Supplementary material for: The Systems Biology Research Tool: evolvable open-source software
Source: BMC Syst Biol. 2008 Jun 29;2:55. doi: 10.1186/1752-0509-2-55 (PMC2446383; doi:10.1186/1752-0509-2-55)
Supplement: Additional file 1 — SBRT Archive. An archive of the current version of the Systems Biology Research Tool. [file 1752-0509-2-55-S1.zip › sbrt-1.4.0/doc/developers_guide/api/sbrt/kernel/math/optimization/package-use.html]

Uses of Package sbrt.kernel.math.optimization


|  |  |  |
| --- | --- | --- |
| **Overview** \| **Package** \| Class \| **Use** \| **Tree** \| **Deprecated** \| **Index** \| **Help** | |  |
| PREV   NEXT | **FRAMES**    **NO FRAMES**     **All Classes** |  |


---


## **Uses of Package sbrt.kernel.math.optimization**

| Packages that use sbrt.kernel.math.optimization | |
| --- | --- |
| **sbrt.ext.cplex** | Provides a class that allows communication with CPLEX. |
| **sbrt.ext.glpk** | Provides a class that allows communication with GLPK. |
| **sbrt.kernel.fba.comp** | Provides classes and interfaces for performing Flux Balance Analysis computations. |
| **sbrt.kernel.fba.proc** | Provides classes and interfaces for executing Flux Balance Analysis processes. |
| **sbrt.kernel.math.optimization** | Provides classes and interfaces for creating objects relevant to solving optimization problems. |
| **sbrt.shell** | Provides classes used by shell-level software. |
| **sbrt.shell.mng.fba** | Provides classes and interfaces for creating Flux Balance Analysis process managers. |
| **sbrt.shell.text.math** | Provides classes and interfaces for defining formats for mathematical objects. |

Classes in sbrt.kernel.math.optimization used by sbrt.ext.cplex

****LinearProgramSolver****
  
          This interface is used to represent linear program solvers.

****OptimizationSense****
  
          This enum type is used to represent the sense, or direction,
of an optimization problem.

****ProgramSolver****
  
          This interface is used to represent program solvers, as in linear
or quadratic program solvers.

****ProgramSolverStatus****
  
          This enum type is used to indicate the status of a program solver.

****QuadraticProgramSolver****
  
          This interface is used to represent quadratic program solvers.

Classes in sbrt.kernel.math.optimization used by sbrt.ext.glpk

****LinearProgramSolver****
  
          This interface is used to represent linear program solvers.

****OptimizationSense****
  
          This enum type is used to represent the sense, or direction,
of an optimization problem.

****ProgramSolver****
  
          This interface is used to represent program solvers, as in linear
or quadratic program solvers.

****ProgramSolverStatus****
  
          This enum type is used to indicate the status of a program solver.

Classes in sbrt.kernel.math.optimization used by sbrt.kernel.fba.comp

****OptimizationSense****
  
          This enum type is used to represent the sense, or direction,
of an optimization problem.

****ProgramSolver****
  
          This interface is used to represent program solvers, as in linear
or quadratic program solvers.

****ProgramSolverStatus****
  
          This enum type is used to indicate the status of a program solver.

Classes in sbrt.kernel.math.optimization used by sbrt.kernel.fba.proc

****OptimizationSense****
  
          This enum type is used to represent the sense, or direction,
of an optimization problem.

Classes in sbrt.kernel.math.optimization used by sbrt.kernel.math.optimization

****OptimizationSense****
  
          This enum type is used to represent the sense, or direction,
of an optimization problem.

****ProgramSolver****
  
          This interface is used to represent program solvers, as in linear
or quadratic program solvers.

****ProgramSolverStatus****
  
          This enum type is used to indicate the status of a program solver.

Classes in sbrt.kernel.math.optimization used by sbrt.shell

****ProgramSolver****
  
          This interface is used to represent program solvers, as in linear
or quadratic program solvers.

****ProgramSolverException****
  
          This class is used to wrap exceptions thrown by program solver packages.

Classes in sbrt.kernel.math.optimization used by sbrt.shell.mng.fba

****OptimizationSense****
  
          This enum type is used to represent the sense, or direction,
of an optimization problem.

****ProgramSolverException****
  
          This class is used to wrap exceptions thrown by program solver packages.

Classes in sbrt.kernel.math.optimization used by sbrt.shell.text.math

****OptimizationSense****
  
          This enum type is used to represent the sense, or direction,
of an optimization problem.

---


|  |  |  |
| --- | --- | --- |
| **Overview** \| **Package** \| Class \| **Use** \| **Tree** \| **Deprecated** \| **Index** \| **Help** | |  |
| PREV   NEXT | **FRAMES**    **NO FRAMES**     **All Classes** |  |


---
